# Supplementary material for: Co-occurrence of beaked whale strandings and naval sonar in the Mariana Islands, Western Pacific
Source: Proc Biol Sci. 2020 Feb 19;287(1921):20200070. doi: 10.1098/rspb.2020.0070 (PMC7062028; doi:10.1098/rspb.2020.0070)
Supplement: Supplemental Table 1 [file rspb20200070supp1.pdf]

Supplementary Table 1. Major International Navy Training Operations in the Mariana Islands Range Complex from 2003 – 2019.

	Exercise Name	Dates	Platforms	Anti-Submarine Operation	Citation
1	Tandem Thrust 2003 *	April 14 – May 5, 2003	Surface ship & aviation	Yes	https://www.navy.mil/submit/display.asp?story_id=7367; https://www.navy.mil/submit/display.asp?story_id=7005; https://www.globalsecurity.org/military/ops/tandem-thrust.htm
2	Valiant Shield 2006	June 19 – 23, 2006	Surface ship & aviation	Yes	https://www.navy.mil/submit/display.asp?story_id=24337; https://www.navy.mil/submit/display.asp?story_id=24249
3	Valiant Shield 2007	August 7 – 14, 2007	Surface ship & aviation	Yes	https://www.navy.mil/submit/display.asp?story_id=31261; https://www.navy.mil/submit/display.asp?story_id=31033
4	Multi-Sail 2010	April 7 – 16, 2010	Surface ship	Yes	https://www.navy.mil/submit/display.asp?story_id=52710
5	Valiant Shield 2010	September 2 – 21, 2010	Surface ship & aviation	Yes	https://www.cpf.navy.mil/news.aspx/000053; https://www.navy.mil/submit/display.asp?story_id=56123
6	US Navy unit level exercise**	August 21, 2011	unknown	Yes	Pers. Comm U.S. Pacific Fleet N465
7	Valiant Shield 2012	September 11 – 19, 2012	Surface ship & aviation	Yes	https://www.public.navy.mil/surfor/Pages/ValiantShield2012Ends.aspx
8	Multi-Sail 2013 *	March 29 – April 5, 2013	Surface ship & aviation	Yes	https://www.public.navy.mil/surfor/ddg82/Pages/StandardMissileLaunch.aspx; https://navylive.dodlive.mil/2013/04/15/your-navy-operating-forward-pacific-ocean-north-arabian-sea-5th-fleet/multi-sail-13-2/
9	Multi-Sail 2014	March, 21 – 26, 2014	Surface ship & aviation	Yes	https://www.navy.mil/submit/display.asp?story_id=79832; https://www.public.navy.mil/surfor/cds-15/Pages/Multi-Sail-2014.aspx
10	GUAMEX 2014	August 9 – 15, 2014	Surface ship & aviation	Yes	https://www.navy.mil/submit/display.asp?story_id=82619, https://navaltoday.com/2014/08/12/guamex-focuses-on-anti-submarine-operations/
11	Valiant Shield 2014	September 15 – 23, 2014	Surface ship & aviation	Yes	https://www.public.navy.mil/surfor/Pages/USPacificCommandForcesComeTogetherforValiantShield2014.aspx; https://www.public.navy.mil/surfor/cds-15/Pages/ValiantShield2014comestosuccessfulend.aspx; https://www.public.navy.mil/surfor/ddg89/Pages/ValiantShieldExerciseKicksOffinPacific.aspx
12	Multi-Sail 2015	March 20 – 30, 2015	Surface ship & aviation	Yes	https://www.public.navy.mil/surfor/cds-15/Pages/desron-15-jmsdf-flex-their-combat-capability-during-multi-sail-2015.aspx
13	GUAMEX 2016	January 22 – 31, 2016	Surface ship	Yes	https://www.public.navy.mil/surfor/cds-15/Pages/US,-Japanese-Ships-Start-Tactical-Exercise-Near-Guam-.aspx

14	Multi-Sail 2016	March 6 – 11, 2016	Surface ship & aviation	Yes	https://www.public.navy.mil/surfor/cds-15/Pages/US-Navy,-JMSDF-Complete-Annual-Multi-Sail-Exercise-.aspx
15	Valiant Shield 2016	September 12 – 23, 2016	Surface ship & aviation	Yes	https://www.public.navy.mil/surfor/Pages/U.S.-Pacific-Command-forces-to-come-together-for-exercise-Valiant-Shield.aspx
16	Multi-Sail 2017	March 6 – 10, 2017	Surface ship	Yes	https://www.public.navy.mil/surfor/cds-15/Pages/U.S.-Navy-JMSDF-complete-annual-MultiSail-exercise-.aspx
17	GUAMEX 2017	July 31 – August 12, 2017	Surface ship & aviation	Yes	https://www.navy.mil/submit/display.asp?story_id=101709
18	Pacific Griffin 2017	August 19 – September 2, 2017	Surface ship	Yes	https://www.public.navy.mil/surfor/lcs4/Pages/US-Singapore-navies-enhance-maritime-partnership-with-training-in-Guam-.aspx
19	Multi-Sail 2018	March 8 – 14, 2018	Surface ship	Yes	https://www.public.navy.mil/surfor/cds-15/Pages/US-Navy-JMSDF-Participate-in-MultiSail-2018.aspx
20	Malabar 2018	June 7 – 10, 2018	Surface ship	Yes	https://www.public.navy.mil/surfor/ccsg5/Pages/US-Indian-and-Japanese-Maritime-Forces-to-Participate-in-Exercise-Malabar-2018.aspx
21	Valiant Shield 2018	September 16 – 26, 2018	Surface ship & aviation	Yes	https://www.public.navy.mil/surfor/ccsg5/Pages/Joint-US-Forces-Team-for-Exercise-Valiant-Shield-2018.aspx
22	Exercise Sea Dragon	January 14 – 26, 2019	Aviation	Yes	https://www.c7f.navy.mil/Media/News/Display/Article/1730836/us-australia-set-to-kick-off-2019-exercise-sea-dragon/

NOTE:
*Dates approximated from U.S. Navy public website
** Not a major international naval operation
