# Supplementary material for: Co-occurrence of beaked whale strandings and naval sonar in the Mariana Islands, Western Pacific
Source: Proc Biol Sci. 2020 Feb 19;287(1921):20200070. doi: 10.1098/rspb.2020.0070 (PMC7062028; doi:10.1098/rspb.2020.0070)
Supplement: Supplemental Table 2 [file rspb20200070supp2.pdf]

Supplementary Table 2. Probability of n stranding events randomly occurring during or within 6 days after documented naval events from June 2006 through January 2019

Number of stranding events associated with naval event	Probability
0	100%
1	40%
2	8%
3	1%
4	0.1%
5	0%
