# Supplementary material for: Co-occurrence of beaked whale strandings and naval sonar in the Mariana Islands, Western Pacific
Source: Proc Biol Sci. 2020 Feb 19;287(1921):20200070. doi: 10.1098/rspb.2020.0070 (PMC7062028; doi:10.1098/rspb.2020.0070)
Supplement: Supplemental Figure S1 [file rspb20200070supp3.pdf]

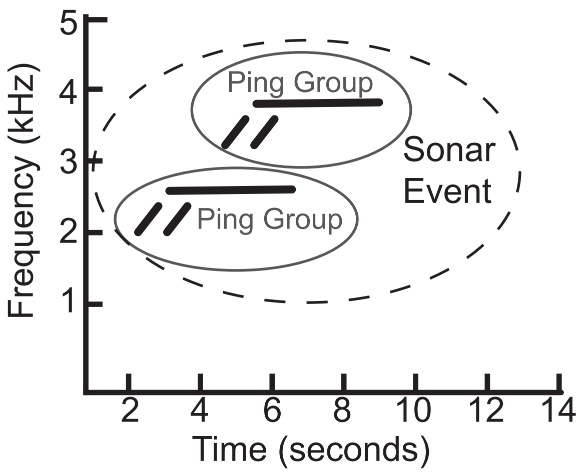

Supplemental Figure S1. Schematic spectrogram shows the grouping definition used by analysts for observed MFAS signals. “Packets” may contain multiple frequency-upsweeps, downsweeps and tonal pulses occurring with gaps of less than 0.1 seconds. A packet may actually consist of two packets that occur within 5 seconds of each other.
