# Supplementary material for: Co-occurrence of beaked whale strandings and naval sonar in the Mariana Islands, Western Pacific
Source: Proc Biol Sci. 2020 Feb 19;287(1921):20200070. doi: 10.1098/rspb.2020.0070 (PMC7062028; doi:10.1098/rspb.2020.0070)
Supplement: Supplemental Figure S2 [file rspb20200070supp4.pdf]

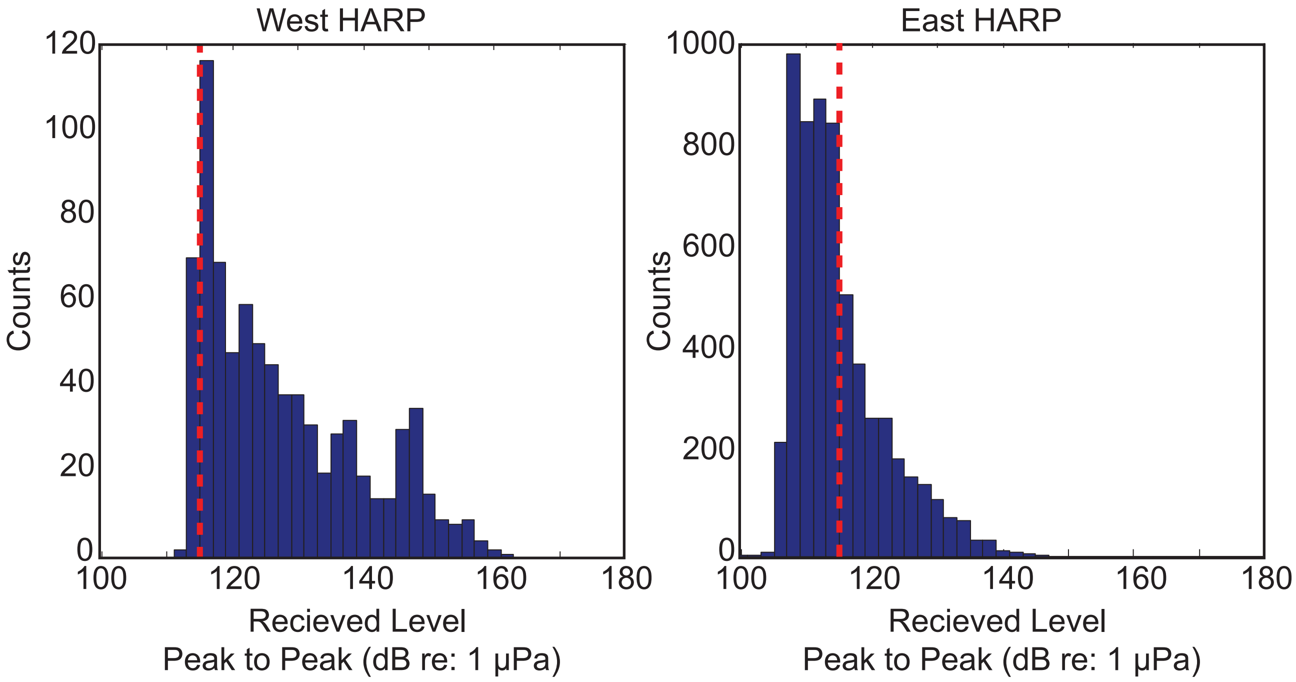

Supplemental Figure S2. Histogram of peak-to-peak received levels of MFAS packets detected by a human analyst at West (left) and East (right) HARPS. A threshold was set at 115 dB re: 1 μPa for reporting acoustic characteristics (duration, RL, SEL) in Table 3.
